# Complete Genome Sequence of *Staphylococcus aureus* Tager 104, a Sequence Type 49 Ancestor

**DOI:** 10.1128/genomeA.00706-13

**Published:** 2013-09-12

**Authors:** Richard Davis, Mohammad J. Hossain, Mark R. Liles, Peter Panizzi

**Affiliations:** Department of Pharmacal Sciences, Harrison School of Pharmacy, Auburn University, Auburn, Alabama, USAa; Department of Biological Sciences, College of Sciences and Mathematics, Auburn University, Auburn, Alabama, USAb

## Abstract

We report here the complete genome sequence of *Staphylococcus aureus* Tager 104, originally isolated from a cutaneous abscess in 1947 by Morris Tager. Sequence typing of the strain revealed its membership in sequence type 49 (ST49), a previously unknown multilocus sequence type (MLST) in clinical samples.

## GENOME ANNOUNCEMENT

Multidrug-resistant strains of *Staphylococcus aureus* pose a significant health threat in both hospital and community settings. Therefore, there is interest in using whole-genome sequencing to gather genomic references for the ancestor strains of current clinical *S. aureus* isolates. In this study, we report the genome sequences obtained for a historical *S. aureus* strain isolated from a cutaneous abscess at the New Haven Hospital in 1947 (1). Herein, the combination of second-generation Illumina MiSeq sequencing technology of high accuracy with third-generation Pacific Biosciences (PacBio) single-molecule real-time (SMRT) sequencing technologies allows for the bridging of complicated genomic regions, such as internal repeats (2). This combination of sequencing technologies, termed hybrid assembly, allows for automated closure of bacterial genomes by newly designed assembly programs.

To generate the genome sequences for strain *S. aureus* Tager 104, genomic DNA was isolated using the EZNA bacterial DNA kit (OMEGA Bio-Tek). Using this DNA, a bar-coded library was constructed using the Nextera DNA sample preparation kit (Illumina), and fragments within the 150-to-750-bp range were isolated over a Size Select-IT kit (OMEGA Bio-Tek). An Illumina MiSeq sequencer was used to generate genome sequences with a 2 × 150 paired-end sequencing kit. This resulted in 2,493,569 paired reads with an average length of 126.7 bp. The sequence reads were trimmed for quality (0.01 quality score) and *de novo* assembled to yield contigs using the CLC Genomics Workbench version 4.6.1. In addition, a genomic sublibrary of strain Tager 104 was produced for PacBio SMRT sequencing technology. Two runs were performed by the Interdisciplinary Center for Biotechnology Research (http://www.biotech.ufl.edu), producing 32,545 and 32,177 filtered reads with average read lengths of 3,310 and 3,301 bp, respectively. These raw PacBio reads were submitted with the raw Illumina reads to the Celera Assembly pipeline of the SMRT analysis 2.0 suite (3). This pipeline produced 239 PacBio contigs that were submitted with the 36 original Illumina contigs to Sequencher 5.1 (using default settings), producing 20 scaffolds. This technique successfully bridged complex genomic regions in the original Illumina contigs, including RNA sequences, which increased from 28 coding regions to 52 coding regions in the final scaffolds.

In order to correct ambiguous bases, scaffolds were uploaded into ChromasPro 1.7.5 (Technelysium Pty., Ltd.), and 40-bp oligonucleotides upstream of the ambiguous bases were copied into the CLC sequence viewer and searched against the raw Illumina reads. Matching reads were uploaded to ChromasPro and aligned to scaffolds using 95% minimum identity and 40-bp minimum overlap. The resulting scaffolds were reordered in progressiveMauve (4) using reference strain *S. aureus* Mu50 (accession no. NC_002758.2), and the output was submitted to RAST for annotation (5). The Tager 104 genome contains 2,868,388 bp, with a G+C content of 31.70%. The genome contains 2,772 open reading frames, including 48 tRNA genes, 4 rRNA operons, and 4 putative genomic islands (6).

The initial characterization of the genome using multilocus sequence typing (MLST) analysis (7) revealed *S. aureus* Tager 104 to be a member of sequence type 49 (ST49), a previously uncharacterized ST type in clinical samples.

### Nucleotide sequence accession numbers.

The complete genome sequence of *S. aureus* Tager 104 has been deposited at DDBJ/EMBL/GenBank under the accession no. AVBR00000000. The version described in this paper is version AVBR01000000.
